# Severe postherpetic neuralgia and facial paralysis in the oral and periauricular regions managed with acupuncture and electroacupuncture: a case report

**DOI:** 10.3389/fpain.2024.1474103

**Published:** 2024-12-23

**Authors:** Junlong Li, Jing Wang, Guiping Li, Jieying Zhang, Boru Zhang, Shu Wang

**Affiliations:** ^1^ First Teaching Hospital of Tianjin University of Traditional Chinese Medicine, Tianjin, China; ^2^ National Clinical Research Center for Chinese Medicine Acupuncture and Moxibustion, Tianjin, China; ^3^ The Second Affiliated Hospital of Tianjin University of Traditional Chinese Medicine, Tianjin, China; ^4^ Tianjin Key Laboratory of Acupuncture and Moxibustion, Tianjin, China; ^5^ Tianjin Academy of Traditional Chinese Medicine Affiliated Hospital, Tianjin, China; ^6^ Key Laboratory of Cerebropathy Acupuncture Therapy of State Administration of Traditional Chinese Medicine, Tianjin, China

**Keywords:** acupuncture, electroacupuncture, postherpetic neuralgia, Ramsay-Hunt syndrome, facial paralysis, case report

## Abstract

Postherpetic neuralgia (PHN) is a severe and persistent pain condition following herpes zoster infection. This case report details the analgesic effects of acupuncture combined with electroacupuncture in a 66-year-old male patient presenting with PHN and peripheral facial paralysis, who showed limited response to conventional treatment with corticosteroids and antiviral medications. Following a comprehensive treatment protocol, including pricking-cupping bloodletting, and targeted acupuncture, the patient experienced significant pain relief and improved facial nerve function. This report highlights the potential of traditional Chinese medicine (TCM) in managing PHN, with sustained improvement observed over a one-year follow-up period.

## 1 Introduction

Ramsay-Hunt syndrome (RHS) is a peripheral neuropathy caused by infection of the geniculate ganglion and adjacent cranial nerves with varicella-zoster virus, also known as herpes zoster virus. The typical symptoms include ear pain, vesicular rash in the ear canal, and ipsilateral facial paralysis (1). Additional symptoms can include dizziness, nausea, hearing loss, and balance disturbances. Acute herpes zoster-related inflammation can lead to nerve fiber degeneration and other structural changes, resulting in spontaneous neural activity that can cause persistent pain lasting more than one month after the resolution of the rash, known as postherpetic neuralgia (PHN) (2). Some patients may continue to experience pain for several months to years, significantly impacting their quality of life. According to recent data, the incidence rates of herpes zoster and PHN in China are 7.7% and 2.3%, respectively, with approximately 29.8% of herpes zoster patients developing PHN (3). This case report details the diagnosis and treatment course of a severe RHS patient primarily presenting with pain symptoms, emphasizing the potential role of traditional Chinese medicine (TCM) in managing PHN.

## 2 Case description

On September 7, 2021, a 66-year-old male patient presented at the acupuncture and moxibustion department of the First Teaching Hospital of Tianjin University of Traditional Chinese Medicine. The patient reported experiencing blisters accompanied by pain in the left oral cavity, face, and scalp for over a month, along with left facial paralysis persisting for one week. The patient was alert and oriented, ambulated independently, responded fluently, and fully cooperated during the physical examination. The body habitus was normal, with no signs of overweight or malnutrition. Vital signs, including body temperature, heart rate, respiratory rate, and blood pressure, were within normal limits.

The patient reported a history of chickenpox during childhood but denied any history of heart disease, immune system disorders, other chronic illnesses, or psychiatric disorders. He also denied a family history of hereditary diseases, as well as any history of drug or food allergies. The patient reported smoking 3–10 cigarettes per day but had no history of regular alcohol consumption.

**Figure 1:**
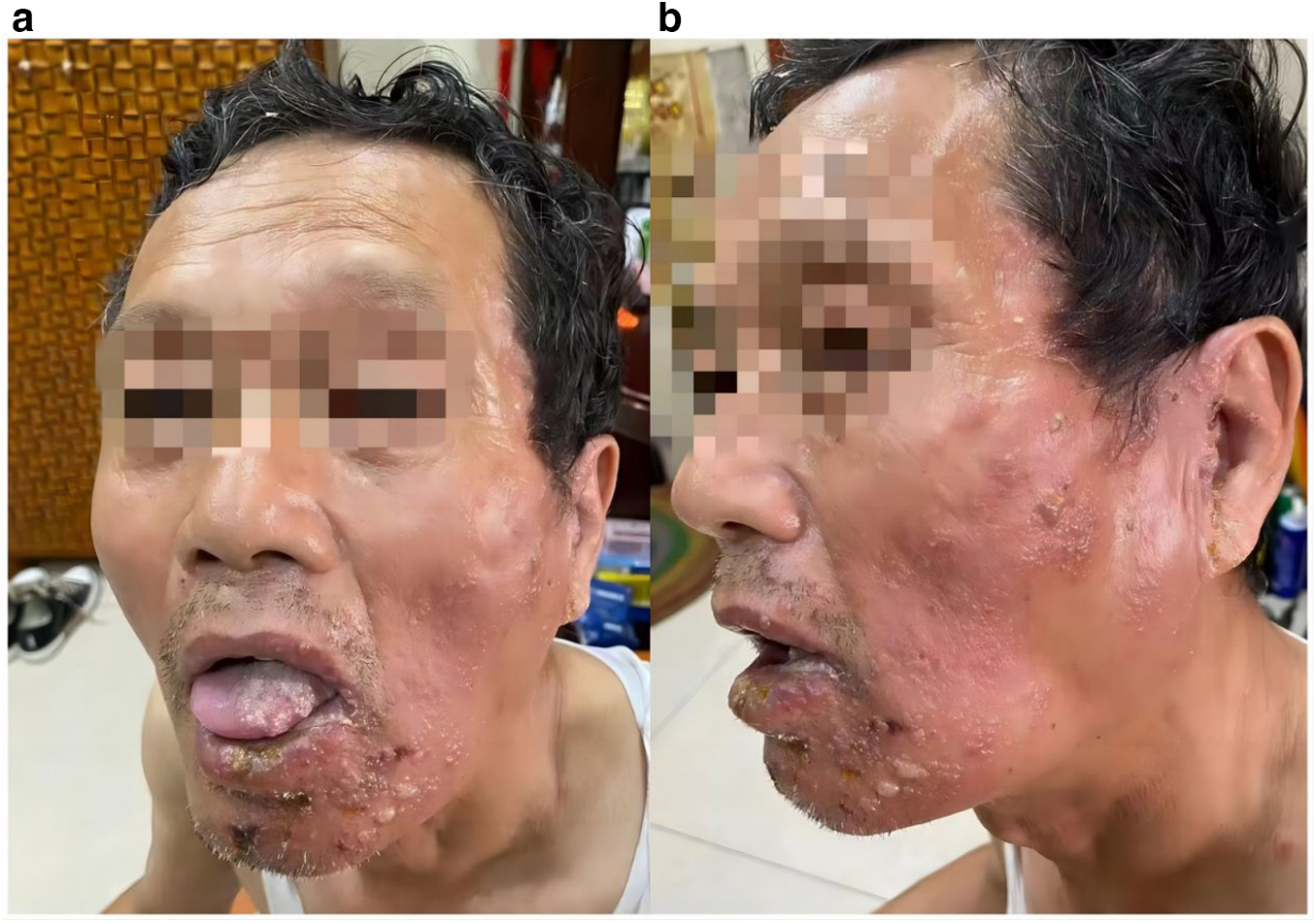
**(a)** Distribution of oral and facial herpes lesions in the patient on the second day of onset. **(b)** Worsening of facial herpes lesions in the patient on the fifth day of onset.

On August 4, 2021, the patient developed intraoral blisters and lesions on the left cheek, accompanied by intense pain and spontaneous toothache in the upper left molar region. Despite self-administering anti-inflammatory and analgesic drugs, pain relief was minimal. He sought medical attention at an oral specialist hospital, where a diagnosis of herpes zoster was made based on scattered blisters and ruptures on his left cheek, lower jaw skin, and mucosa (Figure 1a). An oral surgeon previously performed oral cavity irrigation and medication application under topical anesthesia for the patient. The treatment included dexamethasone sodium phosphate injection, lidocaine hydrochloride injection, vitamin B12 injection, and recombinant human interferon-α2b spray. Additionally, acyclovir gel was applied topically to the skin.

After three days of treatment with no significant pain relief, the patient sought further treatment at a specialized dermatology hospital on August 8th. Symptoms had worsened, with diffuse erythematous patches and clustered vesicles on the left side of the face and ears (Figure 1b). Despite symptomatic treatment with anti-inflammatory, antiviral, immunomodulatory, analgesic, and neurotrophic drugs, pain relief was unsatisfactory. During this period, the patient had used injectable and oral medications, including but not limited to dexamethasone sodium phosphate injection (with a maximum daily dose of 7 mg), foscarnet sodium chloride injection, paracetamol-dihydrocodeine tablets, gabapentin capsules, thymosin enteric-coated tablets, valacyclovir hydrochloride tablets, and mecobalamin tablets. Moreover, the patient developed symptoms of peripheral facial paralysis on the left side. The pain specialist recommended surgical treatment, but the patient opted for traditional Chinese medicine (TCM) at the outpatient department of acupuncture and moxibustion.

Upon examination, the patient exhibited diffuse erythema and edema on the left side of the face and ear, accompanied by blisters and crusted lesions. Notably, left-sided facial paralysis was observed, characterized by a flattened left nasolabial fold, restricted facial movements, and impaired ability to raise the eyebrows and bare the teeth. The patient reported severe, spontaneous burning or electric shock-like pain, predominantly in the left temple and preauricular region. Additionally, significant pain was elicited when the affected area was in contact with clothing, with a Visual Analog Scale (VAS) score of 9–10 and an ID Pain Scale score of 4.

## 3 Treatment

The patient was diagnosed with Ramsay-Hunt syndrome (RHS) by an experienced acupuncturist with over 40 years of practice. Both peripheral facial nerve paralysis and postherpetic neuralgia (PHN) were addressed.

Initially, pricking-cupping bloodletting therapy were applied to the areas where herpes lesions had clustered, mainly around the Taiyang (EX-HN5) and Yifeng (SJ17) points. The therapy involved gently puncturing the local skin with a standard three-edged needle (2 mm × 65 mm) to induce minor bleeding, followed by the application of glass fire cupping (inner diameter 25 mm) to extract local blood. Approximately 5–8 ml of blood was removed during each session.

**Figure 2:**
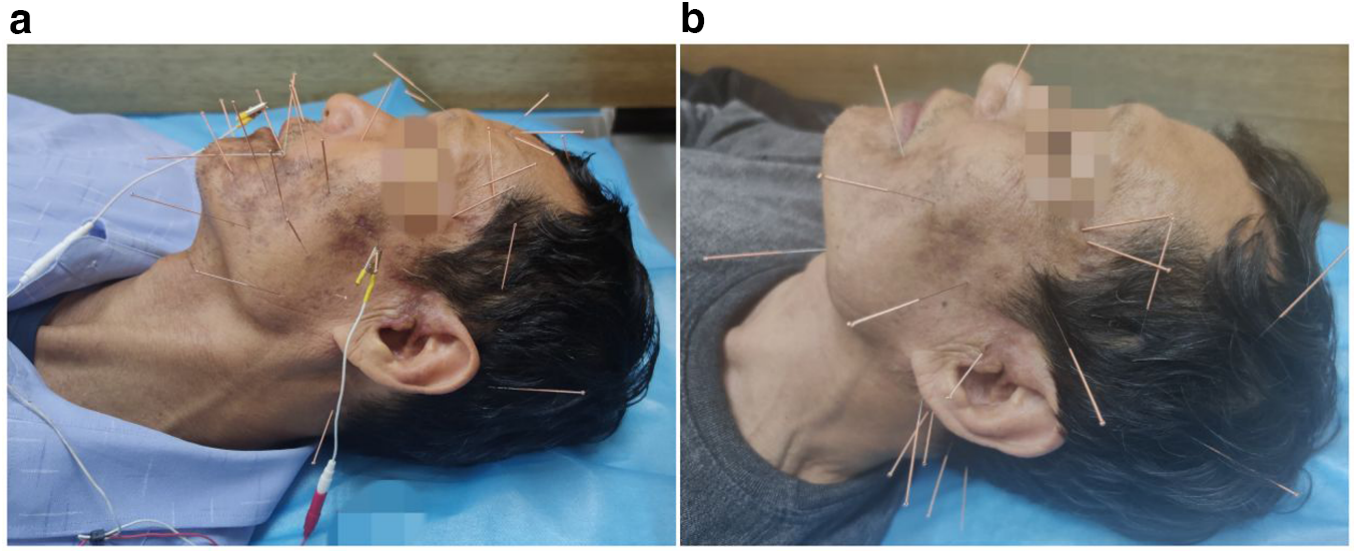
**(a)** Photo taken on the first day of acupuncture treatment, showing scabbing and darkening of some lesions with intense pain sensation. **(b)** Treatment photo taken after nearly two months of acupuncture therapy, with adjustment in acupoint quantity based on recovery progress and discontinuation of electroacupuncture stimulation.

The acupuncture treatment process consisted of four steps:

1. A 75 mm acupuncture needle (Huatuo brand, size 0.25 × 75 mm) was used to puncture Yifeng (SJ17) and Xiaguan (ST7) points at a depth of 45–65 mm, aiming for the deqi sensation.
2. The needle was inserted from the Taiyang (EX-HN5) point towards the Xiaguan (ST7) point, penetrating 45–65 mm, with sensation conduction towards the front of the tragus indicating deqi.
3. Dicang (ST4) was punctured towards Sibai (ST2), Xiaguan (ST7), and Jiache (ST6) in three different directions, with radiating sensations along the needling direction sufficing for the therapeutic effect.
4. 40 mm acupuncture needles (size 0.25 × 40 mm) were used for facial acupoints, including Daying (ST5), Jiache (ST6), Kouheliao (LI19), Yingxiang (LI20), Sibai (ST2), Quanliao (SI18), Cuanzhu (BL2), and Yangbai (GB14), as well as Hegu (LI4) and Waiguan (SJ5) on the limbs. Needles were retained for 30 min after obtaining deqi (Figure 2a).

Electroacupuncture stimulation was applied using a 4HZ/20HZ dense-sparse wave generated by an electric acupuncture device (SDZ-II model by Huatuo) for 30 min, with electrodes connected to Dicang (ST4) and Xiaguan (ST7) points.

The treatment frequency was three sessions per week, with intensity adjusted based on the patient's recovery (Figure 2b).

## 4 Clinical course and outcome

The symptom documentation, evaluation of treatment efficacy, and subsequent follow-up records for this patient were completed by researchers independent of the treating physician.

At the patient's initial visit, the VAS score was 9–10. After one week of treatment, the patient reported noticeable pain reduction, with VAS scores decreasing to 7–8 during pain attacks. Improvement in facial nerve paralysis symptoms was also observed, and all herpes lesions had scabbed over. The patient reported that although episodes remain very painful, he no longer experience pain so severe that it induces the urge to strike his head to relieve it.

Following two months of acupuncture treatment (21 sessions), the pigmented areas on the skin lightened, pain tolerance improved. The VAS score decreased to about 5. Facial nerve paralysis symptoms significantly improved, with restored eyebrow lifting, cheek puffing, and teeth showing functions. The patient reported that although pain persisted, he had been able to resume daily work and activities. Furthermore, with the alleviation of facial nerve paralysis symptoms, he had become more willing to re-engage in normal social interactions with others.

**Figure 3:**
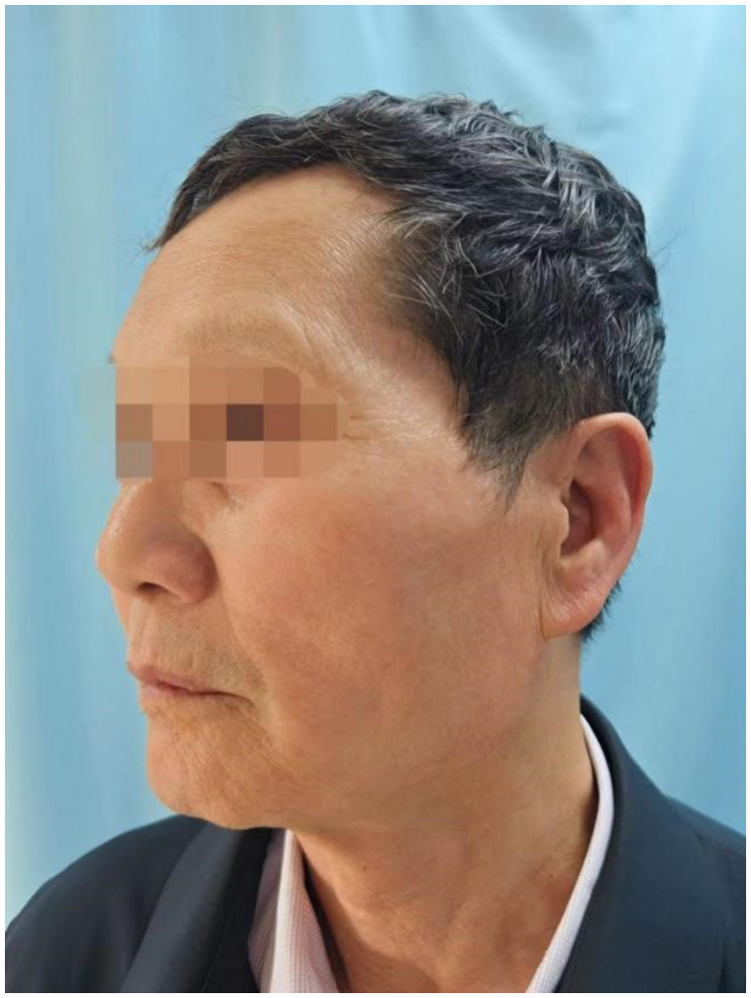
One year later, although the patient still experienced mild facial pain, his condition had improved to the point where it no longer significantly affected daily activities and medication had been discontinued for a period of time.

During a one-year follow-up, the patient occasionally experienced mild, tolerable pain without needing oral medication. The VAS score remained at 3 (Figure 3). The symptoms of facial nerve paralysis fully resolved, with no residual sequelae. The patient mentioned experiencing mild pain only when exposed to hot steam on the face.

In subsequent interviews, the patient mentioned being highly reliant on acupuncture treatment at the beginning of therapy. However, when the VAS score decreased to below 4, the patient began to question whether to continue regular acupuncture sessions. The patient inquired with the treating physician about the possibility of extending the interval between acupuncture sessions or substituting acupuncture with medication. Ultimately, after achieving pain tolerance and resuming daily work and life (approximately 3 months into treatment), the patient completely discontinued acupuncture therapy.

No adverse events or unexpected incidents were reported during the follow-up period.

## 5 Discussion

Current research indicates that a greater extent of neural invasion by the herpes virus (including spinal and cranial nerves) is associated with an increased likelihood of developing postherpetic neuralgia (PHN), characterized by more severe pain. Despite receiving conventional medication for oral herpes zoster infection, this patient exhibited rapid disease progression and widespread infection, resulting in severe PHN and peripheral facial paralysis, indicative of damage to cranial nerves V and VII. In this case, acupuncture points Xiaguan (ST7) and Yifeng (SJ17) were stimulated with relatively strong intensity using longer needles, deeper insertion depth, and electroacupuncture stimulation.

In managing analgesia for PHN, in addition to conventional pharmacological treatments such as calcium channel modulators and tricyclic antidepressants, numerous minimally invasive interventional therapies have proven effective. These include nerve blocks, transcutaneous electrical nerve stimulation (TENS), pulsed radiofrequency (PRF) therapy, and spinal cord and peripheral nerve stimulation (SCS) (4–8).

The analgesic effects of acupuncture are primarily attributed to the stimulation of endogenous opioid release, including endorphins, enkephalins, and dynorphins, which modulate pain perception and provide analgesia. Additionally, acupuncture influences neurotransmitter levels, particularly serotonin and norepinephrine, which are crucial for pain modulation. This therapy also promotes neuroplastic changes that enhance the brain's ability to inhibit pain signals. Furthermore, acupuncture exhibits anti-inflammatory effects by modulating cytokine release, reducing pro-inflammatory and increasing anti-inflammatory cytokines. The electrical stimulation used in electroacupuncture enhances the release of endogenous opioids more effectively than manual acupuncture. It also improves local blood flow, promoting tissue repair and reducing inflammation. The electrical currents used in electroacupuncture modulate pain pathways in both the central and peripheral nervous systems, potentially leading to more significant neuroplastic changes and prolonged pain inhibition (9–13). Additionally, the use of sparse-dense wave electroacupuncture not only alleviates pain but also reduces facial spasms, promotes the improvement of edema, and facilitates the repair of nerve damage (14, 15).

Clinical studies have demonstrated the inhibitory effects of acupuncture on neuropathic pain (12, 16–17), as well as its effectiveness in promoting recovery from facial paralysis caused by facial nerve injury (18). The selected acupoints in this report possess therapeutic effects on both main symptoms of Hunt's syndrome, potentially offering greater benefits compared to separate acupuncture treatments targeting each symptom individually.

In the management of pain disorders through acupuncture, as pain gradually alleviates, patients may perceive the discomfort caused by acupuncture to be comparable to that of their condition, potentially affecting compliance. This discomfort might even hinder the recovery from chronic pain, highlighting a limitation of acupuncture therapy (19). In such instances, integrating alternative therapies like tuina (Chinese therapeutic massage) and acupressure could be considered to potentially enhance prognosis.

## 6 Conclusion

This case suggests that acupuncture and electroacupuncture may offer potential advantages in treating PHN and peripheral facial paralysis associated with shingles-induced RHS. However, the main limitation of this report is its reliance on a single case study. Further validation of these findings will require prospective controlled clinical studies with larger sample sizes. Future research should explore the mechanisms underlying acupuncture's effects on PHN and facial paralysis to optimize treatment protocols.

## Data Availability

The original contributions presented in the study are included in the article/Supplementary Material, further inquiries can be directed to the corresponding author.

## References

[1] Marziali S Picchi E Di Giuliano F Pisani A Mercuri NB Floris R Facial diplegia resembling bilateral Ramsay Hunt Syndrom. J Neurol Sci. (2017) 376:109–11. 10.1016/j.jns.2017.03.011 28431592

[2] Rowbotham MC Davies PS Fields HL. Topical lidocaine gel relieves postherpetic neuralgia. Ann Neurol. (1995) 37(2):246–53. 10.1002/ana.410370216 7847866

[3] Yang F Yu S Fan B Liu Y Chen YX Kudel I The epidemiology of herpes zoster and postherpetic neuralgia in China: results from a cross-sectional study. Pain Ther. (2019) 8(2):249–59. 10.1007/s40122-019-0127-z 31218562 PMC6857181

[4] Huang J Yang S Yang J Sun W Jiang C Zhou J Early treatment with temporary spinal cord stimulation effectively prevents development of postherpetic neuralgia. Pain Physician. (2020) 23(2):E219–30. 32214307

[5] Texakalidis P Tora MS Boulis NM. Neurosurgeons’ armamentarium for the management of refractory postherpetic neuralgia: a systematic literature review. Stereotact Funct Neurosurg. (2019) 97(1):55–65. 10.1159/000499476 30995653

[6] Lin CS Lin YC Lao HC Chen CC. Interventional treatments for postherpetic neuralgia: a systematic review. Pain Physician. (2019) 22(3):209–28. 10.36076/ppj/2019.22.209 31151330

[7] Huang X Ma Y Wang W Guo Y Xu B Ma K. Efficacy and safety of pulsed radiofrequency modulation of thoracic dorsal root ganglion or intercostal nerve on postherpetic neuralgia in aged patients: a retrospective study. BMC Neurol. (2021) 21(1):233. 10.1186/s12883-021-02286-6 34162352 PMC8223296

[8] Isagulyan E Tkachenko V Semenov D Asriyants S Dorokhov E Makashova E The effectiveness of various types of electrical stimulation of the spinal cord for chronic pain in patients with postherpetic neuralgia: a literature review. Pain Res Manag. (2023) 2023:6015680. 10.1155/2023/6015680 37007861 PMC10065853

[9] Wang H Hu Y Deng J Ye Y Huang M Che X A randomised sham-controlled study evaluating rTMS analgesic efficacy for postherpetic neuralgia. Front Neurosci. (2023) 17:1158737. 10.3389/fnins.2023.1158737 37250417 PMC10213647

[10] Liu Q Wu X Guo J Gao J Liu B Wang Y Analgesic effect of electroacupuncture on postherpetic neuralgia: a trial protocol for a multicenter randomized controlled trial. Pain Ther. (2021) 10(2):1755–71. 10.1007/s40122-021-00283-8 34254233 PMC8586289

[11] Wang Y Li W Peng W Zhou J Liu Z. Acupuncture for postherpetic neuralgia: systematic review and meta-analysis. Medicine. (2018) 97(34):e11986. 10.1097/MD.0000000000011986 30142834 PMC6113033

[12] Cui Y Zhou X Li Q Wang D Zhu J Zeng X Efficacy of different acupuncture therapies on postherpetic neuralgia: a Bayesian network meta-analysis. Front Neurosci. (2023) 16:1056102. 10.3389/fnins.2022.1056102 36704010 PMC9871906

[13] Pan L Zeng X Wang G. Early treatment with electroacupuncture at Jiaji acupoints reduce the incidence of postherpetic neuralgia. Asian J Surg. (2024) 47(7):3288–9. 10.1016/j.asjsur.2024.03.152 38609827

[14] Zhang H Chen F. Efficacy of electroacupuncture with sparse-dense-wave on patients suffered acute facial paralysis. Clin Cosmet Investig Dermatol. (2023) 16:1811–9. 10.2147/CCID.S405569 37483469 PMC10361404

[15] Guo Z. Electroacupuncture. In: Wang T Wang W, editors. Acupuncture Techniques. Cham: Springer (2024). p. 291–303. 10.1007/978-3-031-59272-0_18

[16] Li PS Peng XM Niu XX Xu L Ng EH Wang CC Efficacy of acupuncture for endometriosis-associated pain: a multicenter randomized single-blind placebo-controlled trial. Fertil Steril. (2023) 119(5):815–23. 10.1016/j.fertnstert.2023.01.034 36716811

[17] He Y Guo X May BH Zhang AL Liu Y Lu C Clinical evidence for association of acupuncture and acupressure with improved cancer pain: a systematic review and meta-analysis. JAMA Oncol. (2020) 6(2):271–8. 10.1001/jamaoncol.2019.5233 31855257 PMC6990758

[18] Chen N Zhou M He L Zhou D Li N. Acupuncture for Bell's palsy. Cochrane Database Syst Rev. (1996) 2010(8):CD002914. 10.1002/14651858.CD002914.pub5 PMC7133542 20687071

[19] Lee J Napadow V Park K. Pain and sensory detection threshold response to acupuncture is modulated by coping strategy and acupuncture sensation. BMC Complement Altern Med. (2014) 14:324. 10.1186/1472-6882-14-324 25175308 PMC4167271

